# Exploring Climate Niches of Ponderosa Pine (*Pinus ponderosa* Douglas ex Lawson) Haplotypes in the Western United States: Implications for Evolutionary History and Conservation

**DOI:** 10.1371/journal.pone.0151811

**Published:** 2016-03-17

**Authors:** Douglas J. Shinneman, Robert E. Means, Kevin M. Potter, Valerie D. Hipkins

**Affiliations:** 1 U.S. Geological Survey, Forest and Rangeland Ecosystem Science Center, Boise, Idaho, United States of America; 2 Bureau of Land Management Wyoming, Cheyenne, Wyoming, United States of America; 3 Department of Forestry and Environmental Resources, North Carolina State University, Research Triangle Park, North Carolina, United States of America; 4 National Forest Genetics Laboratory, USDA Forest Service, Placerville, California, United States of America; National Cheng-Kung University, TAIWAN

## Abstract

Ponderosa pine (*Pinus ponderosa* Douglas ex Lawson) occupies montane environments throughout western North America, where it is both an ecologically and economically important tree species. A recent study using mitochondrial DNA analysis demonstrated substantial genetic variation among ponderosa pine populations in the western U.S., identifying 10 haplotypes with unique evolutionary lineages that generally correspond spatially with distributions of the Pacific (*P*. *p*. var. *ponderosa*) and Rocky Mountain (*P*. *p*. var. *scopulorum*) varieties. To elucidate the role of climate in shaping the phylogeographic history of ponderosa pine, we used nonparametric multiplicative regression to develop predictive climate niche models for two varieties and 10 haplotypes and to hindcast potential distribution of the varieties during the last glacial maximum (LGM), ~22,000 yr BP. Our climate niche models performed well for the varieties, but haplotype models were constrained in some cases by small datasets and unmeasured microclimate influences. The models suggest strong relationships between genetic lineages and climate. Particularly evident was the role of seasonal precipitation balance in most models, with winter- and summer-dominated precipitation regimes strongly associated with *P*. *p*. vars. *ponderosa* and *scopulorum*, respectively. Indeed, where present-day climate niches overlap between the varieties, introgression of two haplotypes also occurs along a steep clinal divide in western Montana. Reconstructed climate niches for the LGM suggest potentially suitable climate existed for the Pacific variety in the California Floristic province, the Great Basin, and Arizona highlands, while suitable climate for the Rocky Mountain variety may have existed across the southwestern interior highlands. These findings underscore potentially unique phylogeographic origins of modern ponderosa pine evolutionary lineages, including potential adaptations to Pleistocene climates associated with discrete temporary glacial refugia. Our predictive climate niche models may inform strategies for further genetic research (e.g., sampling design) and conservation that promotes haplotype compatibility with projected changes in future climate.

## Introduction

Ponderosa pine (*Pinus ponderosa* Douglas ex Lawson) is the most wide-ranging pine species in North America [1] and is both ecologically and economically important [2]. Ponderosa pine occupies montane environments throughout most of western North America, from southern British Columbia to the U.S.-Mexico border, with scattered populations extending eastward onto the Great Plains [3]. This broad distribution reflects not only the influence of contemporary climate and environmental settings, but also the influence of past climate variability that forced range expansion and contraction and limited some populations to spatially isolated refugia [4]. Climate-induced range shifts combined with topographic isolation likely contributed to intraspecific genetic diversification over time [5], and spatial patterns among both historical and contemporary ponderosa pine populations suggest that genetic divisions occupy well-defined climate niches [4–7]. A clear understanding of the relationships between genetically distinct populations and climate may be key to management and conservation of ponderosa pine under future climate change [6, 8–9].

Two varieties of ponderosa pine are widely accepted, a Pacific variety, *P*. *ponderosa* var. *ponderosa* Laws., and a Rocky Mountain variety, *P*. *ponderosa* var. *scopulorum* Engelm. The Pacific variety is distributed from southern California to British Columbia and extends eastward to Idaho and Montana, while the Rocky Mountain variety occupies the Rocky Mountains from Montana south to Arizona and New Mexico, and extends eastward onto the High Plains. A transition zone between the two varieties occurs near the Continental Divide in west-central Montana [10] along a steep summer-to-winter precipitation gradient that reflects a similarly steep cline in mtDNA haplotype frequencies between *P*. *p*. var. *scopulorum* and var. *ponderosa* [4–5]. At least three further subgroups are commonly proposed within the Pacific variety (Pacific Coast, North Plateau, and Washoe) and two or three for the Rocky Mountain variety (northern Rocky Mountains, Southwestern, and Great Plains) [reviewed by 11–12]. Washoe pine has been treated as a distinct, small-coned species (*P*. *washoenesis* H. Mason & Stockw.) that exists in a handful of high-elevation locations on the western rim of the Great Basin in northeastern California and northwestern Nevada [10–13], but has been determined in several studies not to be highly differentiated from the rest of the species [9, 14–18]. Additionally, *P*. *arizonica* Engelm., a taxon with five-needle fascicles occurring in the Southwestern United States and northern Mexico, was formerly treated as a variety of ponderosa pine, but is now generally considered a separate species because of differentiation of several morphological characteristics [19–21]. It was not included in this study.

Glacial climates of the Pleistocene restricted ponderosa pine to a much narrower geographic distribution than today and may have resulted in isolated refugial locations [4, 22]. However, a sparse paleoecological record of the species during this time period [23–25] makes it difficult to determine the phylogeographic dynamics that contributed to contemporary population distributions. Given this lack of historical information, an important approach for inferring ponderosa pine evolutionary history, especially in terms of how past climates restricted or expanded habitat for the species, is to determine the climate niches associated with extant distributions of evolutionary lineages.

To elucidate these relationships, climate niche modeling is a valuable tool for examining geographical patterns of species in relation to potential climate and environmental predictors [26]. Various statistical methods and machine learning algorithms have been used to generate species bioclimatic niche models, with model selection depending on research needs but also based on whether response variables are represented by presence only, presence-absence, or abundance data [26–27]. For instance, Rehfeldt et al. [28] used a multivariate regression tree analysis (random forest) with presence-absence data to predict climate drivers of nine tree species distributions in the western U.S. (including ponderosa pine). Bioclimatic niche models also have been used increasingly to identify climate profiles among intraspecific genetic divisions, and to provide more realistic range shift projections and relevant information for management and conservation of species under expected climate change [29–30].

Bioclimatic niche research specific to ponderosa pine has demonstrated relatively clear climate boundaries for the species as a whole, as well as among the two broad-scale genetic divisions. Norris et al. [6] used both a classification tree and an ellipsoid model to provide evidence that climate niches among ponderosa pine varieties are primarily shaped by differences in seasonal precipitation, with greater summer- versus winter-dominated precipitation regimes for *P*. *p*. var. *scopulorum* vs. var. *ponderosa*. Rehfeldt et al. [28] used random forest regression tree techniques to accurately predict the spatial distribution of ponderosa pine as a species using predictors based on seasonal and annual temperature and precipitation. Rehfeldt et al. [31] refined this approach to accurately predict the current spatial distribution of the two varieties of ponderosa pine in western North America. Identifying potential climate-genetic distribution relationships may help to explain late-Pleistocene glacial biogeography of ponderosa pine, including potential locations of glacial-age refugia, and can help to identify areas where specific genotypes may be best suited to persist under future climate change conditions. While bioclimatic niche assessments of the two broad varieties of the species have been an initial step toward this objective, they are conducted at a relatively coarse taxonomic scale and are therefore not sufficiently informative at smaller spatial scales. At the same time, wide disagreement exists about the delineation of intra-specific groups within ponderosa pine below this division because of conflicting geographic patterns of morphological characteristics [11–12, 32], which indicate that the species encompasses a complex group of evolutionary units that may be in the process of differentiating into distinct lineages [9, 33–34]. Given the potential of environmental distribution models applied to distinct, molecular-marker-derived genetic lineages within a species to provide more realistic projections of habitat suitability [30], we therefore used the distributions of mtDNA haplotype-defined evolutionary lineages to refine climate-genetic relationships within ponderosa pine.

The distributions of mtDNA haplotypes in pines and other conifers are informative for detecting ancient differentiation events and subsequent dispersal after gene flow has eliminated evidence of different refugial origins using nuclear and chloroplast markers [35–36]. Geographic structure in genetic differentiation is retained longer in mtDNA markers than for chloroplast and nuclear markers because mtDNA is maternally inherited in most pine species and therefore dispersed only by seed movement, rather than by both seed and wind-borne pollen movement, potentially across larger distances [37–40].

A recent study used information from a highly polymorphic mitochondrial DNA minisatellite region to determine the geographic distributions of, and relationships among, evolutionary lineages across the ponderosa pine distribution in the western United States [9]. This research identified 10 distinct mitochondrial DNA haplotypes, and suggested a complex phylogeographic history not formerly known from the other genetic, morphological, or paleoecological research. Potter et al. [9] found a distinct division between five haplotypes detected within the range of the Pacific variety (identified as Haplotypes 1, 5, 8, 9, and 10) and five detected within the Rocky Mountain variety (Haplotypes 2, 3, 4, 6, and 7). This division likely represents both long-term divergence between the Pacific and Rocky Mountain varieties [41] and more recent genetic divergences not well-associated with race, and suggests that unique, historical refugial origins contributed to the modern distribution of the evolutionary lineages. While these mtDNA haplotypes do not have adaptive significance in themselves, they are indicators of long-term biogeographical processes, such as glacial isolation, that may have led to long-term isolation of groups of populations, resulting in differential natural selection in response to different environmental pressures experienced among these groups of populations [4, 9]. Estimating climate niches for the ten recently identified haplotypes could further define the role of climate in shaping ponderosa evolutionary history, identify sampling strategies to better delineate haplotype distributions, and suggest which populations are most likely to be affected by projected changes in future climate.

The primary objectives of this research were five-fold: 1) develop climate niche models for the two ponderosa pine varieties and each of the 10 haplotypes identified by Potter et al. [9]; 2) use these models to predict and map potential climate niches for each haplotype and variety; 3) use these climate niches to reconstruct the potential distribution of the two ponderosa pine varieties during the last glacial maximum (LGM); 4) use the results to assess the role of climate in shaping the phylogeographic and evolutionary history of ponderosa pine; and 5) discuss how this information may aid conservation and management of the species under future climate change. We were able to accomplish these objectives by using a non-parametric predictive modeling approach that provided insights into relationships between ponderosa pine genetic lineages and climate.

## Methods

**Fig 1:**
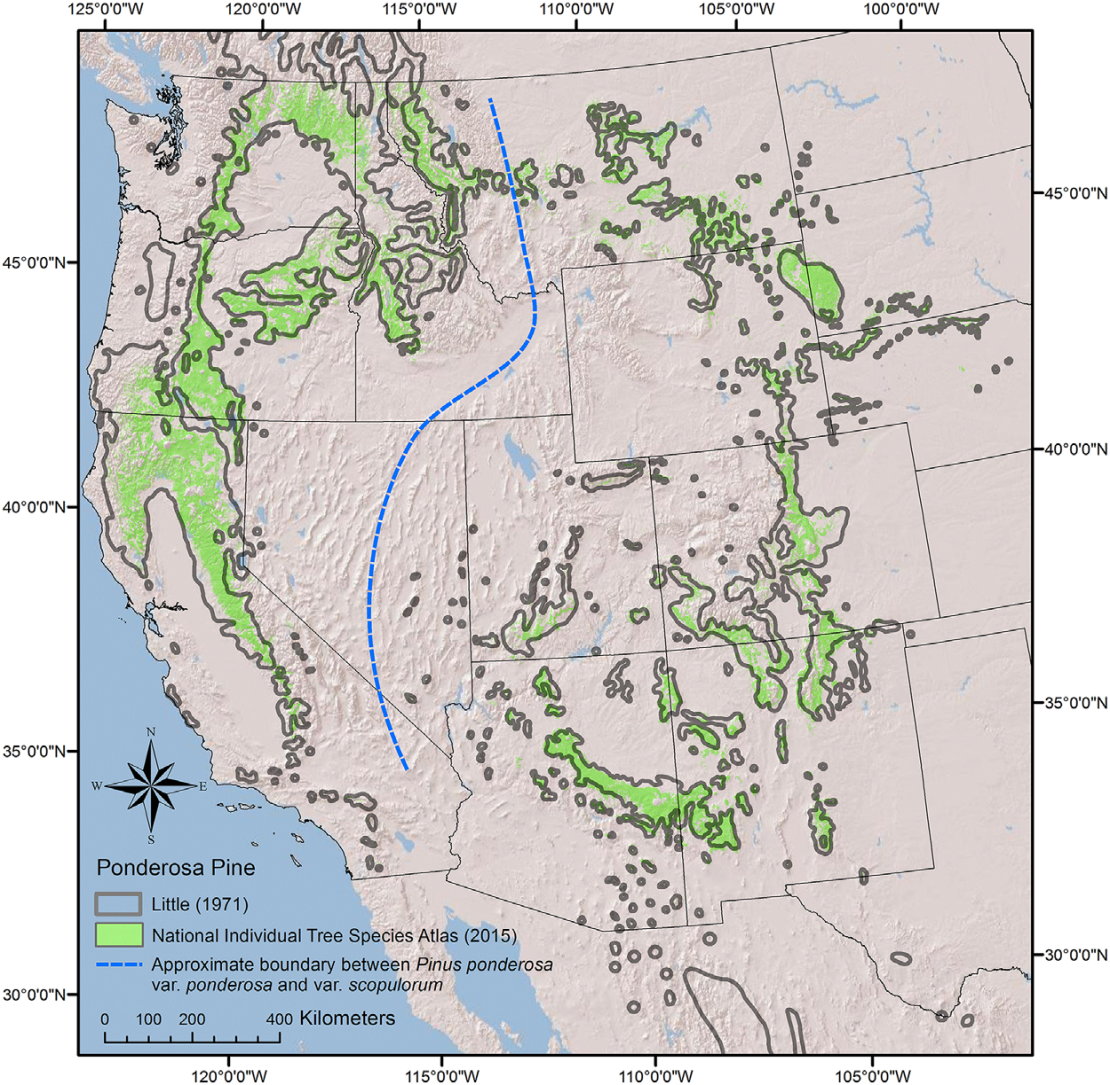
Distribution of ponderosa pine in the western United States based on a recent, fine-scale analysis and classification available from the National Individual Tree Species Atlas (2015) [44] compared to a generalized mapped distribution by Little (1971) [3].

The study area and datasets (described in more detail, below) cover the entire range of ponderosa pine within the 17 western United States (Fig 1). We assessed the potential climate niche of ponderosa pine using four main steps. First, to test the utility of our modeling approach and assess a broad suite of potential climate predictor variables, we used currently available species distribution data to develop overarching climate niche models for the two varieties of *Pinus ponderosa*: var. *scopulorum* and var. *ponderosa*. Second, we used the Potter et al. [9] haplotype dataset to assess the potential climate niche of each individual haplotype. Third, we attempted to refine the climate-based models for each variety and haplotype by adding elevation and topography predictors that might reflect less-well defined spatial correlations between ponderosa pine locations and climate. Finally, we used the resulting statistical models to map estimated distributions of occurrence probabilities for each variety and haplotype.

### The model

We used non-parametric multiplicative regression (NPMR) in HyperNiche v. 2.26 (available: https://www.pcord.com/hyperniche.htm; [42]) to develop climate niche models for the two ponderosa pine varieties and 10 haplotypes. Developing non-parametric models is appropriate given complex relationships among climate predictors and ponderosa pine, including the likelihood of multiplicative effects of predictors, non-linear (often unimodal) haplotype response to climatic variables, and the potential for “multi-niche” space (e.g., bimodal responses). NPMR has been shown to have advantages over other niche modeling methods under these conditions, resulting in increased prediction accuracy and reduced bias [43]. NPMR in HyperNiche analyzes species response as a function of multiplicative interactions among predictors, using a local multiplicative smoothing function and a cross validation procedure to estimate the response variable. An iterative algorithm approach maximizes fit by analyzing target points in a local window (or “neighborhood”) in predictor space via distance-weighted smoothing functions (i.e., kernel smoothing), producing local models that predict the response variable at target points, and repeating this process for all target points to generate a prediction surface (or curve). With presence-absence datasets, the log-likelihood ratio is used to express iterative model improvement (Log*B*) over a “naïve model,” in which the probability of encountering the species is the average frequency of occurrence of the species. NPMR is useful for exploratory analysis [42] by providing nonparametric assessment of probability structures, using a local mean estimator (ensuring estimates of the predicted variable are never outside of its observed range). NPMR can also be used to generate spatially-explicit probability predictions that may indicate where climate-species relationships are relatively strong or where more data may be needed to properly parameterize the model.

### Ponderosa pine variety and haplotype datasets

To estimate the climatological niche of ponderosa pine, we used a geographic information system (GIS) to develop a presence and absence sample of ponderosa pine from 50,000 randomly generated points distributed throughout the study area, with a minimum separation distance of 1 km. Of these, 1920 points occurred where ponderosa pine was most likely present, and these points were paired with a random subsample of 8080 points where it was likely absent (S1 Dataset). To reduce errors of omission and commission in this generated dataset, presence points were selected only if they fell within recently mapped distribution of ponderosa pine forest available in the USDA Forest Service’s National Individual Tree Species Atlas [44] and were also contained within U.S. Geological Survey National Gap Analysis Program vegetation types (http://gapanalysis.usgs.gov) that are dominated by or likely to contain ponderosa pine (S1 Fig). Absence points also required agreement between these two vegetation datasets. Presence points were further delineated as belonging to either *P*. *p*. var. *scopulorum* or var. *ponderosa* based on the distance of each point to sampled haplotype locations, under the assumption that each haplotype is genetically aligned with one of the two varieties [9].

The ponderosa pine haplotype (mtDNA) dataset was derived from foliage samples collected between 2001 and 2012 from a total of 3113 trees representing 104 populations across the range of ponderosa pine within the United States [9] (available at the TreeGene public repository [https://dendrome.ucdavis.edu/tgdr/index.php]; accession # TGDR035, supplemental data). With only two exceptions, at least 20 trees were sampled in each of the 104 populations; most populations encompassed at least 30 sampled trees. Populations were not included in the study if they had undergone reforestation activities. If possible, samples were limited to mature trees and, where feasible, used “legacy” trees and populations that were established before 1900 with no evidence of reforestation planting activities. Sampled trees were at least 100 m apart to increase the probability of sampling the entire range of a population’s genetic composition. Samples were shipped to the National Forest Genetics Laboratory (NFGEL) in Placerville, California, for DNA extraction and sequencing [9].

Due to the spatial clustering of many of the field-sampled locations, the haplotype dataset was filtered for climate niche modeling purposes by selecting location points at least 1 km from each other that also maximized total presence points available for each haplotype NPMR model. NPMR models were constructed for each individual haplotype by treating a known occurrence location for a particular haplotype as a presence, including all haplotype locations associated with the other variety as absences, and then adding 3000 randomly selected non-ponderosa pine absence locations (S2 Dataset). This process resulted in generally small number of presence points (3–45) and different total absence points per haplotype model.

### Climate and spatial predictors

Contemporary climate data were obtained in ASCII-grid format from the U.S. Forest Service, where they were created using thin-plate spline surfaces to interpolate 1961–1990 monthly temperature and precipitation data at ~800 m resolution [45]. Given the likely importance of precipitation seasonality to ponderosa pine distribution [6, 31], we considered predictors that focused either directly on the seasonal measures of precipitation, seasonal precipitation balance (as a ratio), or interactions between temperature and precipitation. We selected 15 climate variables as potential predictors in NPMR candidate models (Table 1). Precipitation variables were log-transformed when this improved normality of distribution, and some climate predictors were combined mathematically (as ratios) if this improved initial model performance. Although some of the climate predictor variables are spatially correlated, we did not preclude their use given the exploratory purpose of this study (i.e., no hypothesis testing). We also included elevation (derived from a 30 arc-second digital elevation model) and a measure of topographic ruggedness [46] as potential predictors, as these attributes can provide microclimates suitable for ponderosa pine [6] that are not captured by the climate datasets. Each variety and haplotype was modeled with the 15 climate predictors and then with the climate and elevation predictors together. Paleoclimate data for ~22,000 yr BP were available at ~ 4km (2.5 minute) spatial resolution from WorldClim (http://www.worldclim.org/paleo-climate), which is derived from CCSM4 hindcasts downscaled and bias corrected using CMIP5 [47]. Using reconstructed paleoclimate monthly temperature and precipitation data, we were able to produce most variables in Table 1, as well as consider other WorldClim “bioclimatic” variables.

**Table 1:** Climate predictor variables used in nonparametric multiplicative regression (NPMR) analysis for ponderosa pine varieties and haplotypes.

Acronym	Definition*
LogMAP	Log10 of mean annual precipitation (MAP)
LogGSP	Log10 of mean growing season precipitation (GSP); Apr–Sep
MTCM	Mean temperature in the coldest month
MTWM	Mean temperature in the warmest month
SUMP	Jul–Aug precipitation
WINP	Nov–Feb precipitation
MMINDD0	Mean minimum degree-days < 0°C
DD5	Degree-days > 5°C
GSDD5	Degree-days > 5°C accumulating in frost-free period
SDAY	Julian date of the last freezing date of spring
PRATIO	GSP/MAP
SDI	Summer moisture index—(√GSDD5)/GSP
SMRPB	Summer precipitation balance: (Jul+Aug+Sep)/(Apr+May+Jun)
SHMI	Summer heat:moisture index—MTWM/Log(Jul-Sept precip)
TDIFF/LogGSP	(MTWM—MTCM)/(LogGSP)

*Temperature is °C and precipitation is mm.

### Model fit, selection, and evaluation

Variables were added to NPMR models in a forward, step-wise procedure. Model fit and selection were generally achieved through a cross-validation procedure, setting a minimum neighborhood size, defining a minimum data-to-predictor ratio, and using a minimum acceptable increase in the log-likelihood ratio (log*B*) with each added variable (as an improvement criterion) to encourage parsimony [42]. Similar to a jackknife approach, NPMR omits each target point to predict its response, forcing predictor selection and associated tolerances to be based on cross-validation results. Log*B* is obtained by dividing the log-likelihood ratio by the number of sample units, and thus is dependent on sample size. We generated models in HyperNiche using a local mean estimator and Gaussian weighting, required a moderate minimum neighborhood size for models (5% of *N*), and restricted model selection to those with a minimum data-to-predictor ratio of 10:1. For the varieties, with large numbers of presence data points, we selected a final model if the addition of a predictor improved Log*B* values by ≥ 2%, and these models were then additionally fine-tuned using decreased minimum average neighborhood sizes for the acceptable models, which allows for more flexible (aggressive) model fitting. In contrast, we used a more conservative approach to encourage parsimony and avoid over-fitting the haplotype models, because of their small numbers of presence points. Adding a predictor to a model required at least 5% improvement in Log*B* values, and selection of a final model with elevation or topography included as predictors required a final Log*B* value at least 5% higher than the best climate-only model. Unlike the variety models, final haplotype models were not fine-tuned for more aggressive model fit. Final variety and haplotype NPMR models were then used to map predicted probability of occurrence values using gridded climate inputs.

To evaluate the fit and predictive success of the final NPMR models for the ponderosa pine varieties, we reevaluated the selected models and original datasets using a leave-one-out, cross-validation procedure that penalizes for overfitting [42]. Several evaluation metrics were generated, including the Area under the Receiver Operating Characteristic curve or “AUC” (area under curve); whereby a maximum AUC value of 1 represents a perfect fit and a value of 0.5 reflects model fit no better than by chance [48]. We also evaluated each model using a pseudo-*R*^2^ (x*R*^2^) value as a relative measure of predictor-response correlation among models; though we recognize the limitations of x*R*^2^ for individually evaluating models that use binary response input data and produce continuous probability outputs (i.e., achieving a x*R*^2^ value of 1.0 is not reasonable) [42]. We also externally-validated model performance using a separate random sample (n = 202 and 298 presence points for vars. *scopulorum* and *ponderosa*, respectively, and 2000 non-ponderosa absence points) that was created using the presence-absence delineation procedures previously described (S3 Dataset). The climate data associated with the presence-absence validation points were input into the selected NPMR model and resulting probability of occurrence predictions were compared to actual occurrences.

The haplotype data presented a greater challenge for validation and model comparison. First, Log*B* is not appropriate for comparing fits among models for different haplotypes, because different numbers of presences influence Log*B* values (i.e., Log*B* increases with more presence values). Second, there was no independent dataset available, and sample sizes were too small to withhold point locations for validation purposes. Therefore, we were not able to externally validate the haplotype NPMR models. However, we used three approaches to judge model performance. First, we assessed the mapped output of occurrence probabilities for each haplotype relative to known ponderosa pine distribution. Second, we calculated mean residual values for predicted presence-absence points. Third, as with the ponderosa pine variety models, we reevaluated each selected model using metrics of model fit and predicative success, including AUC and x*R*^2^.

To reconstruct the climate niche distribution of ponderosa pine during the last glacial maximum (LGM), we modified and adapted the final climate niche models for the two varieties using the reconstructed paleoclimate data. We also added 1605 location points to the *P*. *p*. var. *scopulorum* paleoclimate model to ensure absence point locations included the eastern Great Plains and upper Midwest, given that similarly wetter or cooler climate conditions existed within our primary study area during the LGM [49], and modeling these relationships would be important for accurate hindcasting.

## Results

### Ponderosa pine species-variety climate niches

A single NPMR model for *P*. *p*. var. *ponderosa* and two competing models for *P*. *p*. var. *scopulorum* (with comparable model performance) reliably predicted ponderosa pine occurrence distribution based on the performance of each model (Table 2). Log*B* values indicate better model performance for *P*. *p*. var. *ponderosa* than var. *scopulorum*, but this was due in part to greater number of presence points for the former (see Methods). AUC and x*R*^2^ were quite high for the aggressively tuned variety models (Table 2), and validation of new sites resulted in similarly high AUC values (0.97, 0.97, and 0.99 for var. *scopulorum 1*, var. *scopulorum 2*, and var. *ponderosa* models, respectively), suggesting good predictive ability. Pseudo *R*^2^ (x*R*^2^) values were also relatively high for the validation datasets (0.56, 0.57, and 0.73 for var. *scopulorum 1*, *scopulorum 2*, *and ponderosa* models, respectively), despite the tendency for low x*R*^2^ values for models with binary response variables [42].

**Table 2:** Selected NPMR models for each ponderosa pine (*Pinus ponderosa*) variety and haplotype, with final predictors, response variable sensitivity to predictors (*Q*), evaluation metrics (Log*B*, x*R*², AUC), and number of presence / absence points (see S1 Table for additional model evaluation metric results).

Model	Predictors (*Q*)^1^	Log*B*	x*R*²	AUC	Presence/Absence
var. *ponderosa*	SHMI (0.62) ELEV (0.28) PRATIO (0.21) WINP (0.21)	1165.3	0.78	0.99	1059/8941
var. *scopulorum 1*	PRATIO(0.56) MTWM (0.42) SUMP (0.41) ELEV (0.11)	827.2	0.62	0.98	861/9139
var. *scopulorum 2*	MTWM (0.49) SUMP (0.34) TRIX (0.09) ELEV (0.09)	830.1	0.62	0.98	861/9139
Haplotype 1	SDAY(0.11) SHMI (0.10) PRATIO (0.06) WINP (0.03)	47.6	0.25	0.93	45/3125
Haplotype 2	SMRPB (0.04) TDIFF/LogGSP (0.04)	17.5	0.34	0.78	14/3098
Haplotype 3	LogMAP (0.10) ELEV (0.08) SUMP (0.04)	43.2	0.21	0.95	45/3098
Haplotype 4	PRATIO (0.04) SMRPB (0.04)	14.3	0.14	0.99	9/3098
Haplotype 5	PRATIO (0.06) LogGSP (0.06)	34.1	0.26	0.99	22/3125
Haplotype 6	MTCM (0.12) TDIFF/LogGSP (0.07) WINP (0.02)	23.7	0.06	0.93	34/3098
Haplotype 7	SHMI (0.10) PRATIO (0.07) SMRPB (0.07)	26.3	0.13	0.99	23/3098
Haplotype 8	MTWM (0.08) ELEV (0.06) WINP (0.02)	26.2	0.14	0.96	23/3125
Haplotype 9	ELEV (0.02) PRATIO (0.01)	7.0	0.04	0.83	5/3125
Haplotype 10	PRATIO (0.01) ELEV (0.01)	4.1	0.06	0.99	3/3125

^1^Models with elevation (ELEV) or topographic roughness index (TRIX) as predictors are only shown when Log*B* improved by greater than 5% over the climate-only model. Refer to Table 1 for predictor abbreviation descriptions.

*Q* = sensitivity of response variable after adjusting predictor values (by +/- 5% of their ranges), expressed as the proportion of the range of response variable, and generated by evaluating original “un-tuned” models. Log*B* = log10(likelihood ratio), indicating improvement of a new model over the naive model, presented for un-tuned haplotype models and aggressively tuned variety models. x*R*² = cross-validated *R*² (the cross-validated coefficient of determination, with a maximum of 1.0, and no theoretical minimum), representing evaluation of the un-tuned haplotype models and the aggressively tuned variety models. *AUC* = Area under Receiver Operating Characteristic, calculated for each haplotype model and for the aggressively tuned variety models.

**Fig 2:**
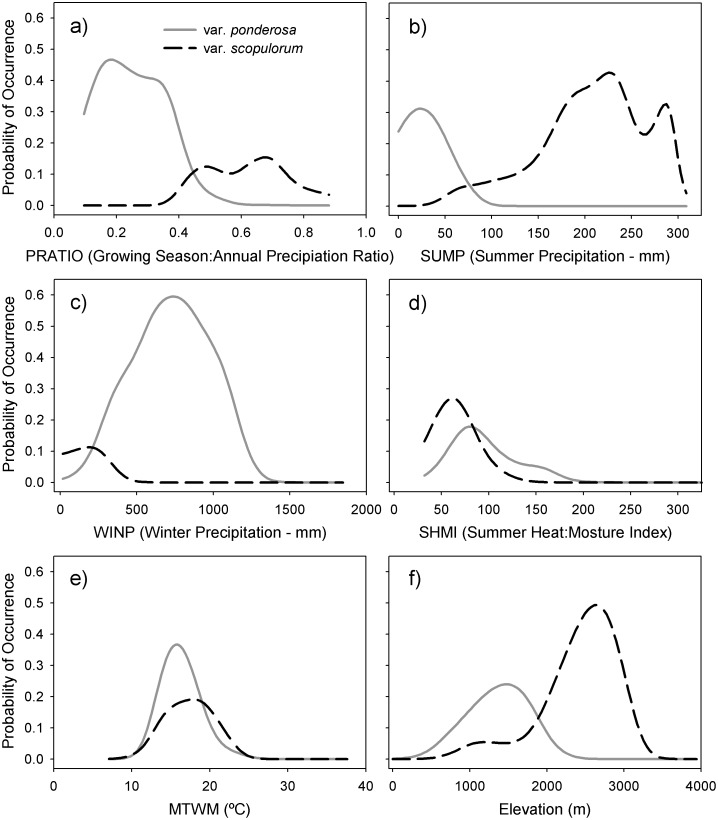
Response curves for *Pinus ponderosa* var. *ponderosa* and var. *scopulorum* in relation to key predictors. Single variable response curves estimating the probability of occurrence distributions for each ponderosa pine variety, using key predictor variables identified in the NPMR models.

The final predictors and mapped distributions reflect fundamental differences in climate niches among the varieties. The final model for var. *ponderosa* included the predictors PRATIO, SHMI, WINP and ELEV, while the two competing models for var. *scopulorum* included: 1) PRATIO, MTWM, SUMP and ELEV; and 2) MTWM, SUMP, ELEV and the topographic roughness index multiplied by converted longitude coordinates (TRIX), which resulted in relatively higher topographic roughness values in the eastern range of the variety (Table 2). To highlight the role of each predictor, two-dimensional NPMR response curves of estimated occurrence probabilities for *P*. *p*. var. *ponderosa* and var. *scopulorum*, respectively, demonstrated distinctive winter- vs. summer-dominated precipitation regimes (Fig 2a), scant vs. ample summer precipitation (Fig 2b), abundant vs. modest winter precipitation (Fig 2c), and higher vs. slightly lower summer heat to moisture ratios (Fig 2d). Responses to summer temperature were similar between the varieties (Fig 2e), as moderate summer temperatures are characteristic over the range of the species, and inclusion of this variable in the full var. *scopulorum* models likely served to separate ponderosa from non-ponderosa point locations. Unique responses to elevation between the varieties indicate a generally high-elevation niche for *P*. *p*. var. *scopulorum* and a mostly medium-elevation niche var. *ponderosa* (Fig 2f).

**Fig 3:**
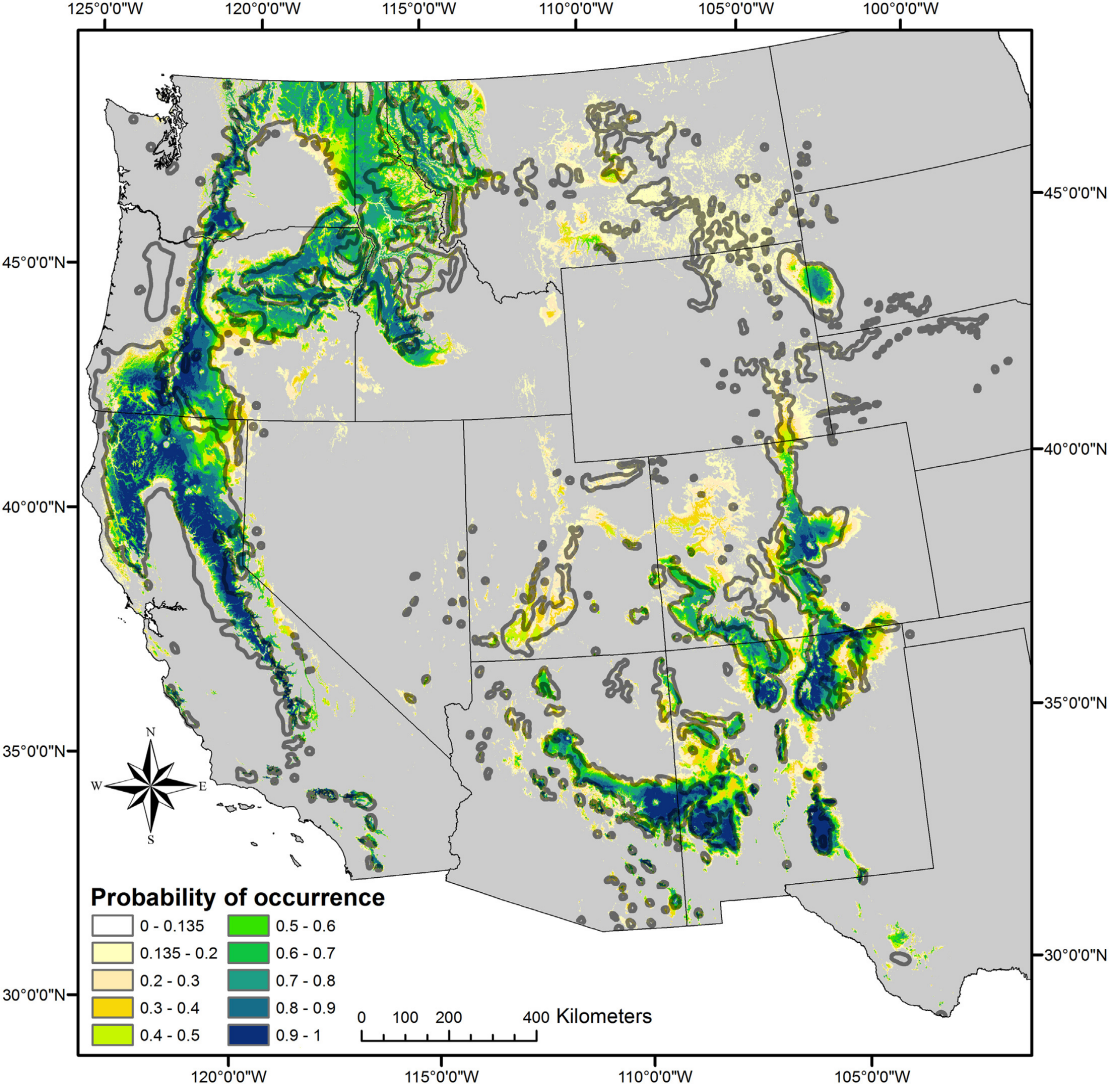
Mapped probability of occurrence predictions for ponderosa pine varieties. Probability of occurrence predictions derived from the NPMR models for var. *scopulorum* and var. *ponderosa* 1 (listed in Table 2), visually combined, with generalized ponderosa pine distribution [3] (shown in gray outline) for context.

The mapped spatial distribution of predicted probability of occurrence values from the climate models was exceptionally good for *P*. *p*. var. *ponderosa* and generally good for var. *scopulorum*, as illustrated in visually-combined, mapped probabilities for the two varieties (Fig 3). Mapped probabilities for *P*. *p*. var. *ponderosa* follow existing distribution maps quite well, with a few minor exceptions. For instance, the model predicted low probability of occurrence values in some mountainous areas in the northern Great Basin, where ponderosa pine populations exist only in a few, isolated locations (e.g., eastern Oregon; [50]). Also, the lack of prediction for *P*. *p*. var. *ponderosa* in the Willamette Valley in western Oregon likely reflects the fact that neither GAP nor USFS classification maps (used to select response points) contained ponderosa pine in this unique climate region, although a few scattered populations exist [51].

Estimated probability of occurrence distributions for models of *P*. *p*. var. *scopulorum* provided excellent spatial correlation with mapped ponderosa pine distributions in the southern half of its range. However, model 1 resulted in generally low probability of occurrence values that were over-extended geographically (map not shown), mostly in the northern Great Plains where both highly-localized and extensive ponderosa pine populations coincide with scattered areas of topographic ruggedness. This relationship was better captured in var. *scopulorum* model 2, but only after experimentally enhancing more easterly topographic roughness values (with longitude values) and by adjusting the lower limits of the mapped probability of occurrence values (to 0.135) to best coincide with known population distributions (Fig 3). Some spatial over-prediction still persisted in the northern Great Plains, and to a lesser extent in northwestern Colorado and north-central Utah, where only scattered, small populations of ponderosa pine exist.

### Haplotype potential climate niches

**Fig 4:**
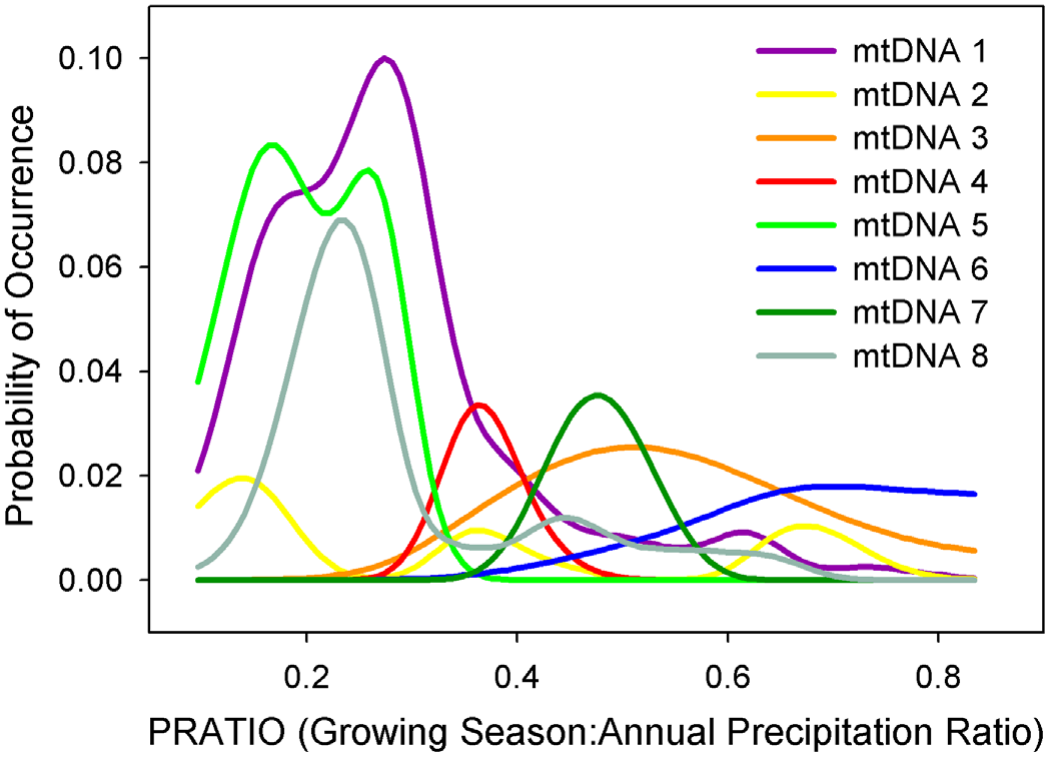
Response curves for ponderosa pine haplotypes predicted by PRATIO. Single variable response curves estimating the probability of occurrence distributions for each haplotype (excluding haplotypes 9 and 10), predicted by the ratio of growing season precipitation to annual precipitation (PRATIO).

In general, Log*B* values for the haplotype NPMR models were small, due to small number of presence points, and x*R*^2^ values were also low but generally increased with Log*B* values (Table 2). Four of the ten haplotype (3, 8, 9 and 10) models were improved after inclusion of ELEV, achieving higher Log*B* and x*R*^2^ values compared to climate only models (Table 2). All final NPMR models for each individual haplotype included at least one seasonal precipitation predictor, with either PRATIO or SMRBP prevalent (Table 2). Based on two-dimensional response curves of estimated occurrence probabilities for each haplotype in relation to PRATIO alone (Fig 4), the peak probability for the three primary haplotypes in *P*. *p*. var. *ponderosa* (1, 5, and 8) occurs when PRATIO is < 0.3, while peak probability for the primary haplotypes associated with *P*. *p*. var. *scopulorum* (3, 4, 6, 7) occurs when PRATIO is between 0.3 and 0.7. The PRATIO predictive influence was also much greater for *P*. *p*. var. *ponderosa* haplotypes (i.e., higher peak probability response values). Haplotype 2, associated with *P*. *p*. var. *scopulorum* but also identified within the range of var. *ponderosa*, has a nearly tri-modal distribution of probability, ranging from one of the lowest to one of the highest PRATIO values among the haplotypes.

**Fig 5:**
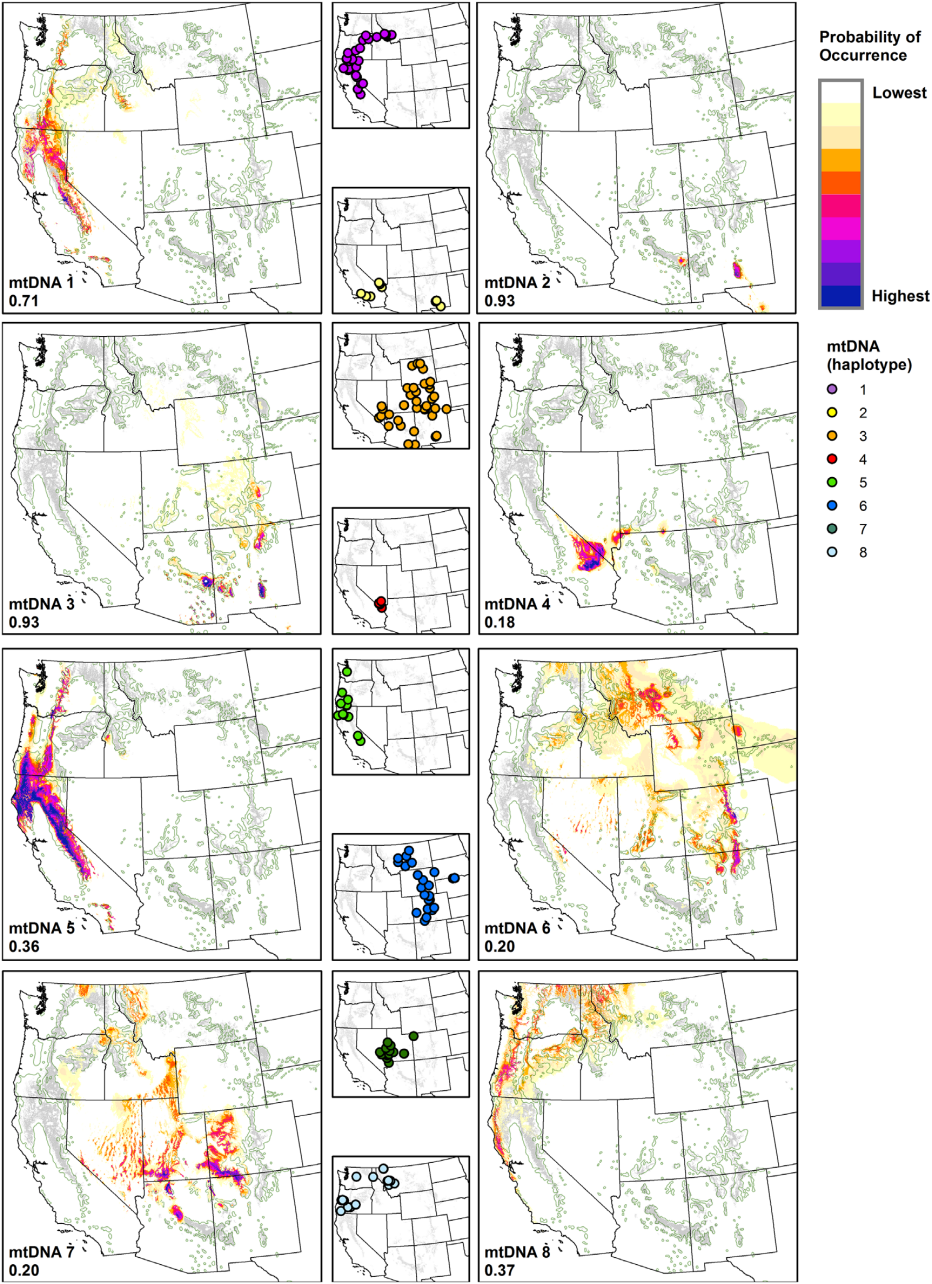
Mapped probability of occurrence predictions for ponderosa pine haplotypes. Probability of occurrence predictions derived from the NPMR models listed in Table 2 for each haplotype. Probability values are color-categorized using 10 equal-interval bins, with maximum probability values for each haplotype provided in the lower left of each map. Haplotypes 9 and 10 were not mapped due to small sample sizes and low Log*B* values. Point locations of sampled ponderosa pine haplotypes are shown in smaller maps. Generalized (green outline) [3] and recently classified (gray shading) [44] distributions of ponderosa pine are shown for context in each map.

Despite mostly low Log*B* values and maximum probability values that varied greatly among NPMR models, mapped estimates of haplotype distributions are generally congruent with known population locations, especially if occurrence probability values are simply classified along a relative gradient of highest to lowest values, basically representing most to least likely climate niches (Fig 5). However, projected estimates of probability of occurrence distributions did not always match known or likely distributions. For instance, similar to var. *scopulorum* model 1, low probability of occurrence values for haplotype 6 were generally geographically over-predicted across much of the northern Great Plains, but also in the central and northern Rocky Mountains. The mapped estimated distributions of haplotypes 4 and 7 were similarly over-predicted relative to extant distributions. The model for haplotype 4, in particular, may reasonably reflect the influence of seasonal precipitation balance on distribution, but it did not incorporate other climatic or topographic predictors that would have further restricted predicted distribution by excluding surrounding Mojave Desert environments. However, inclusion of elevation and topographic roughness did not improve these mapped haplotype predictions. Comparisons of predicted to observed values for each haplotype emphasize that low probability scores were common in most haplotype models, resulting in high mean residuals for the relatively sparse presence points (range among models: 0.72 to 0.95) versus generally low mean residuals for the abundant absence locations (range: -0.001 to -0.011).

### Ponderosa pine distribution during the last glacial maximum

**Fig 6:**
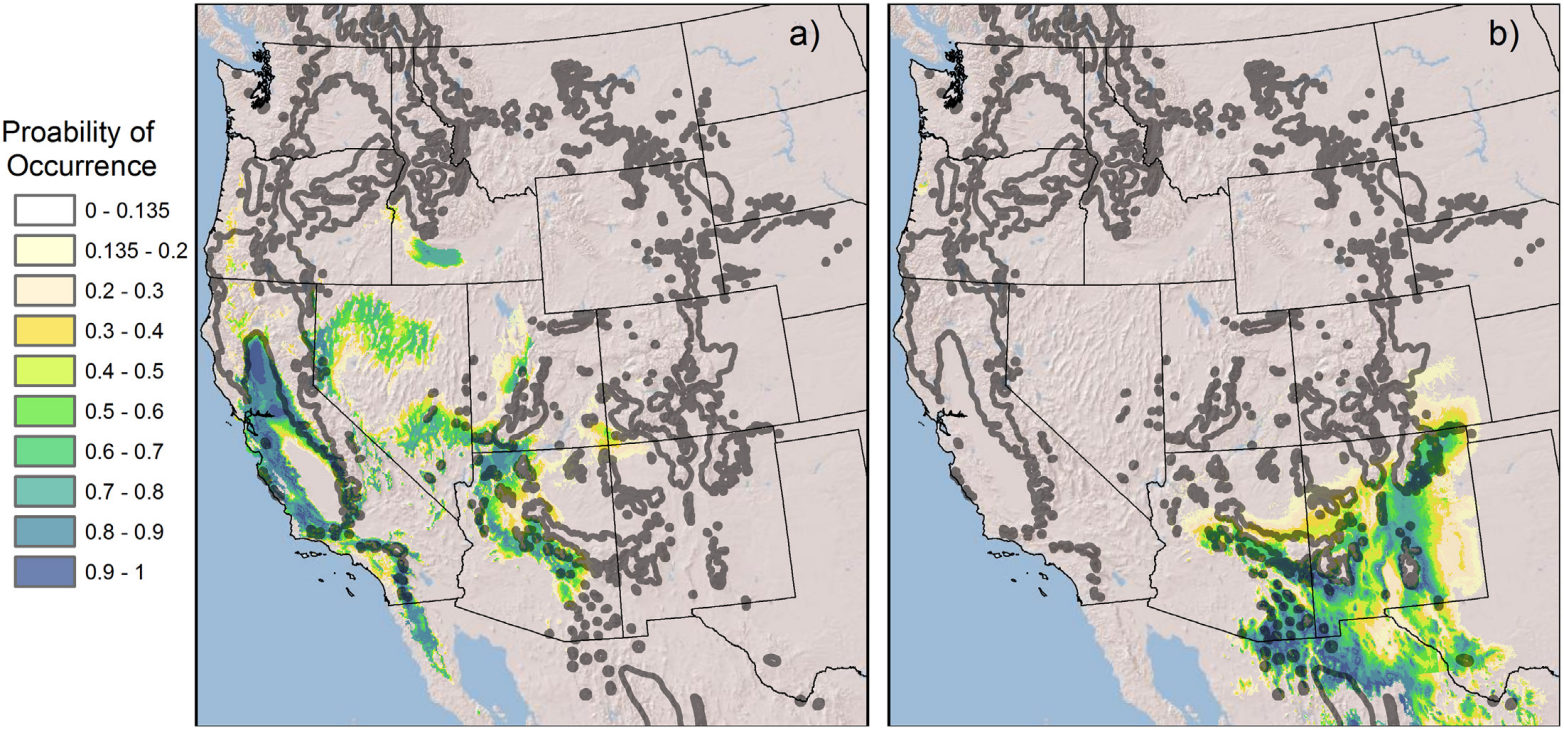
Reconstructed distribution of the potential climate niche for *P*. *p*. var. *ponderosa* (a) and var. *scopulorum* (b) during the last glacial maximum (~22,000 yr BP). Generalized contemporary distribution of ponderosa pine shown in thick gray lines.

We retained all climate variables from the final models for each variety (Table 2) to hindcast climate niche distributions during the LGM. However, because contemporary climate-elevation relationships are not representative of LGM climate, we substituted elevation in each variety model with a temperature-based WorldClim variable [47] that reflected climate differences between the two periods, but also maintained model performance. For the *P*. *ponderosa* vars. *ponderosa* and *scopulorum* models, substituting elevation with mean temperature of warmest month and temperature seasonality, respectively, produced robust though slightly inferior validation results (AUC values of 0.98 and 0.97, respectively) for predicted contemporary climate niches. Potential suitable climate for *P*. *p*. var. *ponderosa* during the LGM was distributed in the lower elevations of the Sierra Nevada, northern Central Valley, southern California coastal ranges, mountains of central and southern Great Basin, mountains and plateaus of northern and central Arizona, the lower Snake River Plain, and scattered lower elevation locations in the Cascade Range, Wasatch Range, and Siskiyou Mountains (Fig 6a). Reconstructed climate niche distribution for *P*. *p*. var. *scopulorum* during the LGM includes the mountain ranges of southern Arizona and New Mexico, lower elevations of the Mogollon Plateau, much of the Rio Grande basin of New Mexico and Texas, and the plateaus and tablelands of northeastern New Mexico (Fig 6b).

## Discussion

### Ponderosa pine climate niches vary with evolutionary lineage

As one of the most widely distributed conifer species in North America, ponderosa pine displays a broad range of tolerance to environmental conditions [32]. However, the species is generally limited to climates with relatively moderate temperature and adequate growing season precipitation [2]. Our results help to clarify important relationships between seasonal precipitation and current distributions of the two ponderosa pine varieties, with growing season to annual precipitation balance and other seasonal precipitation measures serving as important predictor variables in both models (Table 2). *Pinus ponderosa* var. *scopulorum* is generally associated with warmer and wetter summers compared to var. *ponderosa*, which is generally associated with relatively wet winters or early growing seasons and dry summers (Fig 2). Exceptions to these patterns exist, including populations of *P*. *p*. var. *scopulorum* in the far southwest that experience dry late-spring to early-summer months (May and June) and monsoonal precipitation in peak summer months (July-August), and populations of *P*. *p*. var. *ponderosa* in northern Idaho and northeastern Washington where summers are relatively wet [6]. The models for the individual haplotypes provide additional insight into differences in seasonal precipitation balance among populations associated with distinct evolutionary lineages of ponderosa pine, with every model including a seasonal precipitation predictor (Table 2). PRATIO is a convenient and effective single variable to illustrate these relationships; the range of the haplotypes associated with *P*. *p*. var. *ponderosa* can be clearly delineated by winter-dominated precipitation regimes, while haplotypes associated with *P*. *p*. var. *scopulorum* occur in more seasonally-balanced or growing season-dominated precipitation regimes (Fig 4). The exception is haplotype 2, found across the seasonal precipitation spectrum in three widely distributed and potentially disjunct populations.

**Fig 7:**
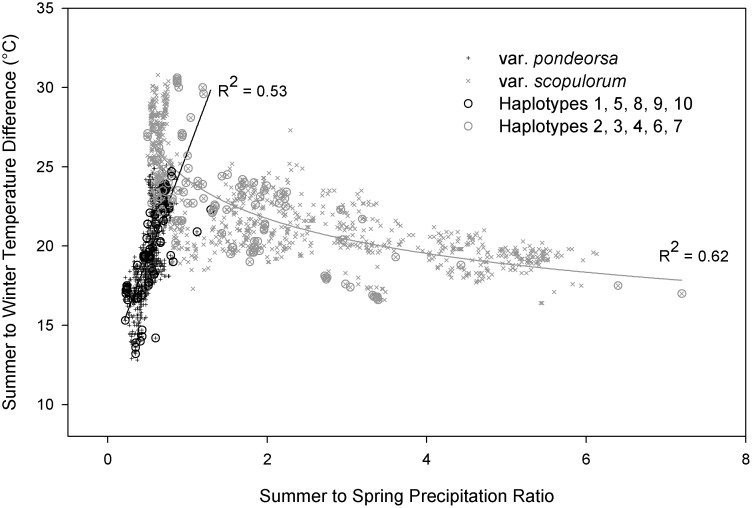
Relationship between seasonal temperature difference and precipitation balance defined for each ponderosa pine variety. Relationship between summer-winter mean temperature difference and summer-spring precipitation balance (ratio) derived from the ponderosa pine variety presence point locations (“x” symbols) and associated haplotype sampled locations (circles). A linear model and power model were fit to the 1961–1990 climate data associated with the *P*. *p*. var. *ponderosa* and var. *scopulorum* locations, respectively, to demonstrate the relative strength of these contrasting climate relationships. Three outlier locations were removed for var. *scopulorum* (Haplotype 2).

The key explanatory climate variables selected in our NPMR models are similar to those identified by Rehfeldt et al. [31], who used random forest regression techniques to identify winter precipitation, summer precipitation, and degree days >5°C as key drivers of climate niche models for ponderosa pine varieties, while important but less influential explanatory variables included warm and cold season degree days, spring moisture, and summer-winter temperature differential. Similar variables were important in our models for the varieties and haplotypes (Table 2), with the notable exception of degree days. Differences in predictor selection among alternative models are not surprising given different methods and datasets used; nevertheless, exploring similarities between models can be insightful. For instance, when our presence locations are graphed along gradients of summer-winter temperature differential (to summarize the range of temperature conditions) versus spring to summer precipitation balance (to summarize the timing of growing season precipitation), distinct variety-climate relationships are apparent (Fig 7). *Pinus ponderosa* var. *ponderosa* locations generally correspond with spring-dominated precipitation regimes, but the proportion of precipitation occurring during summer increases with summer-winter temperature differences. In contrast, *P*. *p*. var. *scopulorum* encompasses a broad range of summer-dominated precipitation regimes, but the proportion of precipitation occurring during spring increases with seasonal temperature differences. Represented this way, the climate niches of the two varieties overlap where there is a relatively even summer-spring moisture balance and moderately-high summer-winter temperature differences. The niche overlap is also apparent among the haplotypes, demonstrating the potential existence of introgression among haplotypes, potentially occurring through long-distance gene flow via wind-dispersed pollen. When occurrence probabilities are estimated from annual precipitation ratios alone, the lower tails of the probability distributions for both haplotypes 1 and 8 extend into growing season precipitation dominance characteristic of haplotypes associated with *P*. *p*. var. *scopulorum* (Fig 4). This climate niche overlap coincides geographically with the introgression of the two varieties along a steep clinal divide located in west-central Montana [4, 18], where a steep but spatially complex gradient occurs between winter- and growing season-dominated precipitation regimes, and provides a unique juxtaposition of continuous habitats among northeastern-most populations of *P*. *p*. var. *ponderosa* and northwestern-most populations of var. *scopulorum*. Indeed, Potter et al. [18] determined that the microsatellite genetic composition of three populations of *P*. *p*. var. *scopulorum* in this region was more closely associated with genetic clusters identified with var. *ponderosa*.

### Model limitations and strategies for refining future haplotype sampling efforts

Climate niche models can lead to poor predictive power of fundamental species-climate-environment relationships, especially if models rely solely on contemporary species distributions not in equilibrium with their environment, ignore spatial autocorrelation issues and geographic proximity effects, or overlook potentially important predictor variables [26, 52]. Although our NPMR haplotype models yielded distinct climate relationships among haplotype population locations (e.g., Fig 4), we consider these models as exploratory, intended to suggest key climate relationships and help direct future research. Hence, we did not limit model results by converting continuous probability of occurrence estimates into presence-absence predictions using an arbitrary threshold value. A few key imitations of this approach, as well as additional insights, are discussed here.

The spatially-clustered, stand-level sampling locations that provided haplotype presence data were effective and efficient for delineating haplotype genetic structure across the broad range of ponderosa pine [9], but this sample design resulted in a spatially filtered and restricted sample size for niche modeling purposes (see Methods). Thus, inconsistent distribution predictions are probably due in part to sparse sample sizes. However, because our NPMR models included controls for over-fitting, which prevented overly complex relationships and restrictive estimates of occurrence probabilities, we were able to glean important information from them. In particular, when small sample sizes coincided with broad spatial extent over diverse habitat conditions (e.g., haplotype 6), spatially projected probability values were generally low and over-dispersed in key locations. In contrast, small sample sizes that coincided with spatially-restricted populations (e.g., haplotypes 2, 4, 7) yielded spatial over-prediction of higher probability values, though some “over” predicted niche space for these haplotypes might represent occupied habitat (that should be sampled for confirmation) or viable but unoccupied habitat due to dispersal limitations across extensive desert landscapes. A systematic resampling design within populations across potential haplotype ranges and direct incorporation of spatial autocorrelation as a model term might improve upon future climate niche predictions [53–54]. Estimated haplotype distributions may also reflect inadequate predictor information (e.g., microclimates) or endogenous factors (e.g., dispersal limitations relative to isolated mountain habitat). The inclusion of elevation in some of our models, and topographic roughness in var. *scopulorum* model 2, point to the influence of topography on temperature and moisture conditions (especially in the Great Plains, where ponderosa pine is typically limited to breaks, steep valleys, and buttes [55]) beyond that reflected in the climate data used. If computationally feasible, future modeling efforts should consider using fine-scale topographic-microclimate inputs. Finally, because elevation was included in our models for the varieties (Table 2), and elevation-climate relationships varied over geologic time, we needed to substitute this predictor variable to be able to credibly hindcast the climate niche distributions during the LGM (Fig 6).

### Implications for evolutionary history

Recent examinations of patterns of ponderosa pine genetic diversity and inferred evolutionary relationships support the idea of climatologically distinct refugial locations and unique evolutionary histories [9, 18], and expands on traditional classification of two primary varieties (*P*. *p*. vars. *ponderosa* and *scopulorum*) and four to six races [4, 5]. Our climate niche modeling for the varieties and haplotypes provides additional information to infer potential phylogeographic processes that shaped recent evolutionary history of ponderosa pine and the development of its evolutionary lineages. Our modeling results are particularly valuable, because we utilized recently available mitochondrial DNA (mtDNA) haplotype data [9] that represent the first range-wide ponderosa pine genetics assessment using molecular data. Moreover, mtDNA may better reflect earlier refugial locations, because it is dispersed only by seed movement, and gene flow is generally more restricted compared to chloroplast DNA (cpDNA), which is broadly dispersed via wind-borne-pollen in conifers [40]. Thus, while mtDNA haplotypes do not have adaptive significance by themselves, they are indicators of long-term biogeographic processes, such as isolation in glacial refugia that resulted in the evolution of separate lineages with putatively different adaptations to the environmental conditions to which they were exposed [56]. Analyzing the current and past environmental niches of these haplotypes additionally offers the potential for a finer temporal scale assessment of biogeographic processes in ponderosa pine than does focusing only on the two varieties of the species.

Previous genetic research has suggested that *P*. *p*. vars. *ponderosa* and *scopulorum* may have been separated for more than 250,000 years [41], long before the last glacial maximum. While initial differentiation of some of the *P*. *p*. var. *ponderosa* haplotypes likely predates the Quaternary, more recent glaciation cycles of the Pleistocene are thought to have induced further genetic diversification by intermittently restricting ranges, likely including localized refugia [22], followed by subsequent migration, hybridization and introgression [33].

Modeled climate niches during the LGM largely reinforce the idea of relatively distinct geographic distribution for the two ponderosa pine varieties at the end of the Pleistocene, with the climate niche for *P*. *p*. var. *ponderosa* largely restricted to the Sierra Nevada, California coastal ranges, and portions of the Great Basin, while the climate niche for var. *scopulorum* was restricted to the southwestern interior highlands (Fig 6). However, our models also suggest potential overlap of climate niches for the varieties in central Arizona. The reconstructed LGM climate niches for ponderosa pine largely correspond with regions that had cool temperate climates with generally wetter than current conditions [49]. Roberts and Hamann [57] used species distribution models (SDMs) to reconstruct climate niches for ponderosa pine during the LGM, and their results are generally similar to ours. However, among other methodological differences, Roberts and Hamann [57] used occurrence data for the entire ponderosa pine species, rather than separating data by variety. In addition, they predicted a much smaller area of LGM climate suitable for ponderosa pine in the California Floristic Region compared to our results, and a relatively high probability of ponderosa pine presence onto the southern and central Great Plains, which our models did not. Despite general agreement among these models, macrofossil evidence for LGM populations in most of these regions is lacking, suggesting that either: a) suitable climate may have existed that went unoccupied by ponderosa pine due to dispersal limitations caused by inhospitable climate; b) some LGM refugia for ponderosa pine remain undetected; or c) our models require refinement.

It is possible that glacial refugia for *P*. *p*. var. *ponderosa* occurred in the southern Sierra Nevada where fossil records have been dated to at least 45,000 yr BP [58], and from the unglaciated Klamath-Siskiyou region in northern California and Oregon [56]. This may explain the current broad distributions of haplotypes 1, 5, and 8, which are found in Klamath-Siskiyou, Cascade, Sierra Nevada, and northern Rocky Mountain locations, while more recent mutations may explain the highly restricted ranges of haplotypes 9 and 10 [9]. The likelihood of a long history of ponderosa pine evolutionary history in the California Floristic Region [59], combined with a strong winter-dominated precipitation regime associated with *P*. *p*. var. *ponderosa* haplotypes, suggest a long evolutionary history of adaptation to winter-precipitation-dominated climates of the Pacific coastal region. The ranges of haplotypes 1 and 8 suggest potential environmental limitations of this part of the ponderosa pine evolutionary tree, as they abruptly end where summer-precipitation dominance begins (Fig 5).

Glacial refugia for *P*. *p*. var. s*copulorum* are more difficult to determine, in part due to sparse paleoecological data, but this variety was thought to have been absent during the last glacial maximum from the central and northern Rockies, Great Basin, Mojave Desert, and Colorado Plateau [23, 60], largely matching the hindcast climate niche distribution for the variety (Fig 6), possibly due to drier and shorter growing seasons [61, 62]. The potentially more ancient lineage of haplotype 7 (possibly sister to all other haplotypes) and its isolated habitats in mountain ranges of the Great Basin and the northern Colorado Plateau suggest these regions could have provided refugia pre-dating the end of the Pleistocene [9, 63], though no fossil evidence has been found to support this theory. Seasonal precipitation predictors were key drivers of the climate model for haplotype 7, which occupies an intermediate winter-summer seasonal precipitation climate niche compared to other haplotypes associated with *P*. *p*. var. *scopulorum*. Haplotype 2 was found in climatically and geographically disjunct locations in southern California, southern Nevada, and southern New Mexico, and has the most complex relationship with seasonal precipitation patterns (Fig 4), suggesting possible expansion from refugia in the Sierra Madre Occidental of Mexico [9, 62] or remnants of diverse southern refugial locations [64]. Indeed, high probability of contemporary occurrence values were predicted for haplotype 2 in the Sierra Madre and Sierra de la Baja California of Mexico (results not shown). Haplotype 4, which is found in southern Nevada and co-occurs with haplotype 2, may be a relatively recent mutational divergence representing genetic differentiation in populations that are uniquely adapted to winter-spring dominated precipitation. Haplotype 6, evolutionarily intermediate between haplotypes 7 and 3, could have emerged from multiple glacial refugial populations on the Great Plains and Rocky Mountains [9]. The highest probability of occurrence values for haplotype 6 were estimated for the Colorado Rocky Mountains, where genetic diversity suggests glacial refugia may have existed [18]. Haplotype 6 ranges as far north as northern Wyoming and south central Montana, and extends to the Black Hills and onto the northern Great Plains in the Upper Missouri River Basin, where it may have arrived from ~6000 to less than 1000 yr BP in certain locations [6, 65–66]. This recent migration into the northern Great Plains was likely facilitated in part by scattered, topographic-microclimates, where our models were over-dispersed but generally predicted low probability of occurrence (Fig 5). Pleistocene populations of Haplotype 3 were likely separated from haplotype 6 by the Rocky Mountains during glacial periods, and refugial locations may have occurred in Arizona and New Mexico [9], where our models now predicted the highest probability of occurrence for the haplotype, in summer monsoon-dominated climates. Norris et al. [7] reconstructed the northward expansion of *P*. *p*. var. *scopulorum* during the late Holocene using dated macrofossil records and suggested that haplotypes 3 and 6 likely responded to northward and westward increases in summer temperature and rainfall.

Finally, the broadly distributed ranges of some haplotypes across climatically diverse regions (especially for haplotypes 1, 3, 6, and 8), corroborates genetic evidence that multiple localized refugia may have persisted until the end of the Pleistocene, leading to more recent local expansion. Potter et al. [18] used microsatellite molecular marker analysis and isozyme analysis to assess genetic variation across the range of ponderosa pine, and suggested that refugia may have existed farther north than southern New Mexico and Arizona. This hypothesis is supported by the idea that contemporary populations located closer to Pleistocene refugia should have more genetic variation than those colonized more recently [67]. Potter et al. [18] discovered ponderosa pine locations with consistently high allelic richness or more unique alleles (relative the general population) in southern Arizona/New Mexico, southern Nevada/Utah, north-central Colorado, and northern California/southern Oregon, and they speculated that local refugia may have been maintained in topographically suitable microhabitats during glaciation. Although these potential refugial locations are not supported by fossil evidence, they may help to explain more recent and rapid mid- to late late-Holocene expansion of haplotypes into disparate climate regions.

### Implications for management and conservation

Given expected changes in future climate under higher carbon emission scenarios [68], it is not surprising that species distribution models project substantial shifts in the future ranges of many forest species [28]. Maintaining ponderosa pine on western North American landscapes will require consideration of genetic variability and suitability under future climates. Rehfeldt et al. [31] used an ensemble of climate models to project a ~ 50% reduction in the range of *P*. *p*. var. *scopulorum* by 2060, including significant areas on the Mogollon Rim, Black Hills and in eastern Montana, where ponderosa pine is currently the predominant forest tree species. In contrast, Rehfeldt et al. [31] projected offsetting habitat losses and gains for *P*. *p*. var. *ponderosa*, which has greater potential for both northward and higher-elevation expansion. In light of such modeled projections, Potter et al. [18] used their recent range-wide analysis of genetic variation and structure in ponderosa pine to suggest that separate gene conservation efforts may be required for the two varieties, including a focus on conserving areas with high genetic variation, unique alleles, and rare gene pools that might preserve adaptive potential under unique future climate conditions.

Although bioclimatic modeling has limitations, including estimating direct demographic response, it is useful for the practical task of guiding climate change adaptation strategies for forest management activities including reforestation, conservation actions, or habitat restoration [69]. For instance, climate-altered disturbance regimes, including wildfire, drought, and biological disturbance agents, are likely to act as key drivers of rapid ecological change in plant communities under future climate [70–71]. Dodson and Root [72] examined stand-replacing wildfires in Oregon forests along an elevation gradient, and found that ponderosa pine may not be able to successfully regenerate in its currently occupied lower elevation range, because soil moisture under current climate was insufficient for spring growth of the seedling taproot in local genotypes. Understanding how to manage post-disturbance landscapes will be critical for maintaining sustainable and resilient ecosystems in the future. Determining viable planting stock for restoration may need to consider the advantages of using genotypes from local versus off-site populations, to best match anticipated long term changes in climate and growth environments. An important management strategy and research opportunity may include planting different haplotypes following large-scale disturbances, taking into consideration their fast-evolving bioclimatic spaces under climate change.

Delineation of the potential climate niches of different ponderosa pine varieties and haplotypes provides yet another source of information to assist managers in the future management of ponderosa pine. In particular, defining these niches may provide insight into managing ponderosa pine genetic variability to encourage the best fit on local landscapes under expected future climate conditions. Scientists and land managers may want to focus particularly on areas with high potential for range contraction, to consider which haplotypes may be best suited for future niches at those sites, given current climate-haplotype relationships. This information may also inform new approaches or suggest focused additions to existing research (e.g., provenance studies), in order to better test the suitability of genotypes to altered climates in high risk areas.

## Supporting Information

S1 Dataset *Pinus ponderosa* presence and absence locational dataset used to produce the NPMR climate niche models for var. *ponderosa* and var. *scopulorum*. For each record, the variety (Variety) is provided (“P” = var. *ponderosa*, *“*S” = var. *scopulorum*, “-” = non ponderosa pine locations), followed by longitude and latitude. Additional absence locations used for LGM reconstructions begin after the 10,000^th^ record.
(CSV)

S2 Dataset *Pinus ponderosa* presence and absence locational dataset used to produce the NPMR climate niche models for the 10 haplotypes identified by Potter et al (2013). For each record, the haplotype number is provided (1–10, with 0 = non-ponderosa pine location), followed by longitude and latitude. The “Exclude” field indicates the haplotype models for which that that record was not used as an absence, because the haplotype number being modeled shared a location (within the same population as) the haplotype number(s) indicated. The original ponderosa pine genetic data used to derive these locations are available at the TreeGene public repository (https://dendrome.ucdavis.edu/tgdr/index.php); accession # TGDR035 (supplemental data).
(CSV)

S3 Dataset *Pinus ponderosa* presence and absence locational dataset used to validate the NPMR climate niche models for var. *ponderosa* and var. *scopulorum*. For each record, the variety (Variety) is provided (“P” = var. *ponderosa*, *“*S” = var. *scopulorum*, “-” = non ponderosa pine locations), followed by longitude and latitude.
(CSV)

S1 Fig Map of the *Pinus ponderosa* presence and absence points used to produce climate niche models for var. *ponderosa* and var. *scopulorum*. (DOCX)

S1 Table Additional metrics for evaluating model fit and predictive success. Selected models were reevaluated using a “leave-one-out cross validation” procedure that penalizes for overfitting [42].
(DOCX)
