# Supplementary material for: Exploring Climate Niches of Ponderosa Pine (Pinus ponderosa Douglas ex Lawson) Haplotypes in the Western United States: Implications for Evolutionary History and Conservation
Source: PLoS One. 2016 Mar 17;11(3):e0151811. doi: 10.1371/journal.pone.0151811 (PMC4795796; doi:10.1371/journal.pone.0151811)
Supplement: S1 Fig — (DOCX) [file pone.0151811.s004.docx]

**Supplementary information for:**

**Exploring climate niches of ponderosa pine (*Pinus ponderosa* Douglas ex Lawson) haplotypes in the western United States: implications for evolutionary history and conservation**

Douglas J. Shinneman^1^, Robert E. Means^2^, Kevin M. Potter^3^, and Valerie D. Hipkins^4^

^1^U.S. Geological Survey, Forest and Rangeland Ecosystem Science Center, Boise, ID, United States of America

^2^Bureau of Land Management Wyoming, Cheyenne, Wyoming United States of America

^3^Department of Forestry and Environmental Resources, North Carolina State University, Research Triangle Park, North Carolina, United States of America

^4^National Forest Genetics Laboratory, USDA Forest Service, Placerville, California, United States of America

*Corresponding author email: [dshinneman@usgs.gov](mailto:dshinneman@usgs.gov)


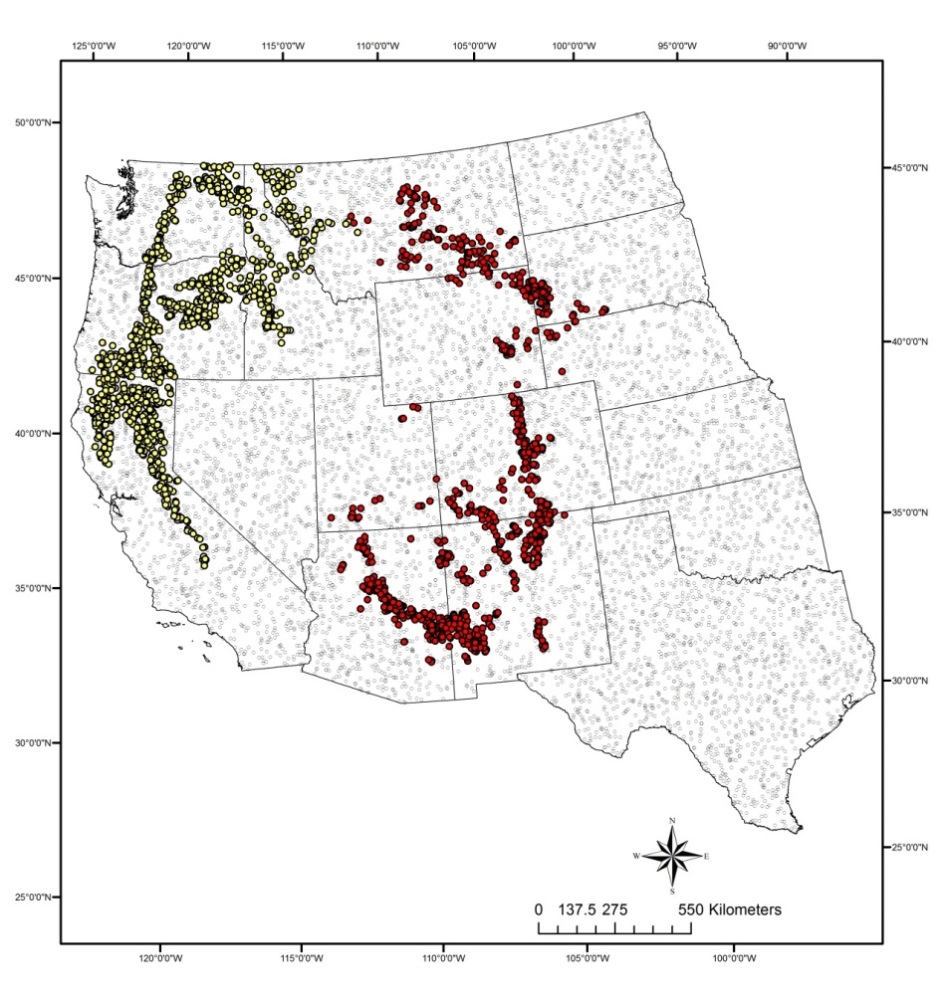


**Figure S1**. **Map of the *Pinus ponderosa* presence and absence points used to produce climate niche models for var. *ponderosa* and var. *scopulorum***. Var. *ponderosa* points are shown as yellow circles (n = 1059), var. *scopulorum* as red circles (n = 861), and absence points as smaller hollow circles (n = 8080). Data-points were generated using a random selection of points > 1 km distance from each other. Ponderosa pine presence and absence points required spatial agreement between two sources: 1) a recent U.S. Forest Service ponderosa pine distribution map (Ellenwood JR, Krist FJ, Romero SA. National Individual Tree Species Atlas. FHTET-15–01. Fort Collins, Colorado: USDA Forest Service, Forest Health Technology Enterprise Team; 2015) that was produced using 30-meter Landsat satellite data, climate, topography, and soil predictors, as well as USFS Forest Inventory and Analysis plot data; and 2) U.S. Geological Survey National Gap Analysis Program (GAP) land cover types (http://gapanalysis.usgs.gov/gaplandcover/data/download) that likely or potentially contained ponderosa pine. The following GAP Land Cover ecosystem types were included (with Level 3 Codes): Mediterranean California Lower Montane Black Oak-Conifer Forest and Woodland (4317), Madrean Pinyon-Juniper Woodland (4518), Madrean Pine-Oak Forest and Woodland (4315), East Cascades Oak-Ponderosa Pine Forest and Woodland (4550), Mediterranean California Mixed Evergreen Forest (4320), East Cascades Mesic Montane Mixed-Conifer Forest and Woodland (4602), Northern Rocky Mountain Dry-Mesic Montane Mixed Conifer Forest (4524), Northern Rocky Mountain Foothill Conifer Wooded Steppe (5302), Northern Rocky Mountain Ponderosa Pine Woodland and Savanna (4529), Northern Rocky Mountain Western Larch Savanna (4510), Northwestern Great Plains - Black Hills Ponderosa Pine Woodland and Savanna (4548), Rocky Mountain Poor-Site Lodgepole Pine Forest (4544), Rocky Mountain Subalpine-Montane Limber-Bristlecone Pine Woodland (4532), Southern Rocky Mountain Dry-Mesic Montane Mixed Conifer Forest and Woodland (4528), Southern Rocky Mountain Mesic Montane Mixed Conifer Forest and Woodland (4610), Southern Rocky Mountain Ponderosa Pine Savanna (5605), Southern Rocky Mountain Ponderosa Pine Woodland (4530), California Montane Jeffrey Pine-(Ponderosa Pine) Woodland (4520), Mediterranean California Dry-Mesic Mixed Conifer Forest and Woodland (4519), Mediterranean California Mesic Mixed Conifer Forest and Woodland (4603), Sierran-Intermontane Desert Western White Pine-White Fir Woodland (4546), Columbia Plateau Western Juniper Woodland and Savanna (4513), Columbia Basin Foothill Riparian Woodland and Shrubland (9831), Inter-Mountain Basins Montane Riparian Systems (9855), Rocky Mountain Lower Montane Riparian Woodland and Shrubland (9825), Northern Rocky Mountain Montane-Foothill Deciduous Shrubland (5808), Southern Rocky Mountain Montane-Subalpine Grassland (7206), Rocky Mountain Lower Montane-Foothill Shrubland (5806), Willamette Valley Upland Prairie and Savanna (5505), Apacherian-Chihuahuan Semi-Desert Grassland and Steppe (5303), Central California Coast Ranges Cliff and Canyon (3213), North Pacific Serpentine Barren (3404).
