# Supplementary material for: Exploring Climate Niches of Ponderosa Pine (Pinus ponderosa Douglas ex Lawson) Haplotypes in the Western United States: Implications for Evolutionary History and Conservation
Source: PLoS One. 2016 Mar 17;11(3):e0151811. doi: 10.1371/journal.pone.0151811 (PMC4795796; doi:10.1371/journal.pone.0151811)
Supplement: S1 Table — Selected models were reevaluated using a “leave-one-out cross validation” procedure that penalizes for overfitting [42]. (DOCX) [file pone.0151811.s005.docx]

**Supplementary information for:**

**Exploring climate niches of ponderosa pine (*Pinus ponderosa* Douglas ex Lawson) haplotypes in the western United States: implications for evolutionary history and conservation**

Douglas J. Shinneman^1^, Robert E. Means^2^, Kevin M. Potter^3^, and Valerie D. Hipkins^4^

^1^U.S. Geological Survey, Forest and Rangeland Ecosystem Science Center, Boise, ID, United States of America

^2^Bureau of Land Management Wyoming, Cheyenne, Wyoming United States of America

^3^Department of Forestry and Environmental Resources, North Carolina State University, Research Triangle Park, North Carolina, United States of America

^4^National Forest Genetics Laboratory, USDA Forest Service, Placerville, California, United States of America

*Corresponding author email: [dshinneman@usgs.gov](mailto:dshinneman@usgs.gov)

|  | Cross Validation | | | | | Overall | | | | | Improved | | Not Improved | | Improvement | |  |  |  |  |
| --- | --- | --- | --- | --- | --- | --- | --- | --- | --- | --- | --- | --- | --- | --- | --- | --- | --- | --- | --- | --- |
| Model  Name | SST | SSE | xR2 | COR | AUC | Pres-  ent | Abs-  ent | Naïve  p | Est. p>  naïve | Est. p<  naïve | P | A | Omi-ssion | Comm-ission | % | Odds  Ratio | logB | aveB | ChiSq | p |
| *pond.* | 947 | 207 | 0.782 | 0.885 | 0.99127 | 1059 | 8935 | 0.106 | 1557 | 8437 | 1052 | 8430 | 7 | 505 | 94.9 | 18.52 | 1165.27 | 1.308 | 5366.25 | 0 |
| *scop1* | 787 | 299 | 0.619 | 0.79 | 0.97984 | 861 | 9138 | 0.0861 | 2094 | 7905 | 835 | 7879 | 26 | 1259 | 87.1 | 6.78 | 827.24 | 1.21 | 3809.6 | 0 |
| *scop2* | 787 | 300 | 0.618 | 0.79 | 0.98255 | 861 | 9135 | 0.0861 | 1972 | 8024 | 841 | 8004 | 20 | 1131 | 88.5 | 7.68 | 830.07 | 1.211 | 3822.6 | 0 |
| Hap1 | 44.4 | 33.2 | 0.251 | 0.509 | 0.93218 | 45 | 3123 | 0.0142 | 481 | 2687 | 41 | 2683 | 4 | 440 | 86 | 6.14 | 47.6 | 1.035 | 219.2 | 0 |
| Hap2 | 13.9 | 9.23 | 0.338 | 0.641 | 0.78002 | 14 | 3097 | 0.0045 | 456 | 2655 | 10 | 2651 | 4 | 446 | 85.5 | 5.91 | 17.54 | 1.013 | 80.79 | 0 |
| Hap3 | 44.4 | 35.2 | 0.207 | 0.467 | 0.95352 | 45 | 3098 | 0.0143 | 570 | 2573 | 43 | 2571 | 2 | 527 | 83.2 | 4.94 | 43.22 | 1.032 | 199.01 | 0 |
| Hap4 | 8.97 | 7.75 | 0.136 | 0.447 | 0.9943 | 9 | 3097 | 0.0029 | 338 | 2768 | 9 | 2768 | 0 | 329 | 89.4 | 8.44 | 14.25 | 1.011 | 65.63 | 0 |
| Hap5 | 21.8 | 16.2 | 0.258 | 0.509 | 0.99125 | 22 | 3125 | 0.007 | 169 | 2978 | 22 | 2978 | 0 | 147 | 95.3 | 20.41 | 34.09 | 1.025 | 157 | 0 |
| Hap6 | 33.6 | 31.6 | 0.059 | 0.255 | 0.93434 | 34 | 3097 | 0.0109 | 838 | 2293 | 34 | 2293 | 0 | 804 | 74.3 | 2.89 | 23.74 | 1.018 | 109.32 | 0 |
| Hap7 | 22.8 | 19.9 | 0.127 | 0.395 | 0.98526 | 23 | 3088 | 0.0074 | 550 | 2561 | 23 | 2561 | 0 | 527 | 83.1 | 4.9 | 26.27 | 1.02 | 121 | 0 |
| Hap8 | 22.8 | 19.7 | 0.139 | 0.384 | 0.95562 | 23 | 3125 | 0.0073 | 370 | 2778 | 23 | 2778 | 0 | 347 | 89 | 8.07 | 26.22 | 1.019 | 120.74 | 0 |
| Hap9 | 4.99 | 4.8 | 0.039 | 0.199 | 0.98656 | 5 | 3125 | 0.0016 | 231 | 2899 | 5 | 2899 | 0 | 226 | 92.8 | 12.85 | 6.99 | 1.005 | 32.21 | 0 |
| Hap10 | 3 | 2.83 | 0.057 | 0.248 | 0.82976 | 3 | 3125 | 0.001 | 149 | 2979 | 3 | 2979 | 0 | 146 | 95.3 | 20.42 | 4.09 | 1.003 | 18.84 | 0 |

**Table S1**. **Additional metrics generated for evaluating model fit and predictive success.** Selected models were reevaluated using a “leave-one-out cross validation” procedure that penalizes for overfitting [42].

**NOTES:**

Sample units with empty neighborhoods were excluded from these calculations.

SST = total sum of squares

SSE = error sum of squares

xR² = cross-validated pseudo-R-squared. -99.9999= missing if < 2 nonempty neighborhoods.

COR = Pearson correlation between 0/1 data and continuous estimate of probability.

AUC = Area Under Curve for Receiver Operating Characteristic (ROC) curve.

Overall present = number of "present" cases (non-zero or exceeding binary cutoff value).

Overall absent = number of "absent" cases (zero or less than or equal to binary cutoff value.)

Naïve p = naive estimate of the probability of occurrence = proportion present.

Est p>naïve = number of cases predicted more likely than average (naive p) to be "present"

Est p< naïve = number of cases predicted less likely than average (naive p) to be "present"

Improved P = number of "present" cases with estimated probability > naive p.

Improved A = number of "absent" cases with estimated probability < naive p.

Not improved omission = Error of omission: present but est.p <= naive p

Not improved commission = Error of commission: absent but est.p >= naive p

Improvement % = Percent of cases with estimates improved over naive model.

Improvement odds ratio = Percent of cases with estimates improved over naive model.

Odds of improvement = proportion of improved/(1 - proportion improved) 9999.9=inf.

logB = log10(likelihood ratio)

aveB = average contribution of each case to the likelihood ratio = 10^(logB/N)

ChiSq = 2*ln(B) = Deviance comparing model to naive model

p = probability of type I error from chi-square distribution, d.f.=1
